# Bioremediation of crude oil and heavy metal pollutants in marine environment by biosurfactant, rhamnolipid isolated from S*tutzerimonas stutzeri −* MW15

**DOI:** 10.1016/j.jgeb.2025.100507

**Published:** 2025-05-29

**Authors:** J.B. Sony, W.A. Manjusha, V.S. Sangeetha, Salom Gnana Thanga Vincent, T. Citarasu, J.R. Anusha

**Affiliations:** a Department of Biotechnology, Malankara Catholic College, Mariagiri, Kaliyakavila, Affiliated to Manonmaniam Sundaranar University, Abishekapatti, Tirunelveli 627012 Tamil Nadu, India; b Department of Biotechnology, Noorul Islam Arts and Science College, Kumaracoil, Tamil Nadu, India; c Department of Environmental Science, University of Kerala, Trivandrum 695581 Kerala, India; d Aquatic Animal Health Laboratory, Centre for Marine Science and Technology (CMST), Manonmaniam Sundaranar University, Rajakkamangalam, Kanyakumari District 629502 Tamil Nadu, India

**Keywords:** Biosurfactant, Bacteria, *Stutzerimonas stutzeri*, Rhamnolipid, Bioremediation

## Abstract

**Objectives:**

The current research aimed to study the biodegradation potential of biosurfactants isolated from marine bacteria against crude oil and heavy metals.

**Methods:**

Hemolytic activity, oil displacement, drop collapse, tilted glass slide, and emulsification index tests were employed for screening the biosurfactant production efficiency of marine bacteria which was cultured on an enrichment mineral medium. Based on the highest emulsification activity, the most effective bacterial isolates were selected and subjected to biosurfactant isolation. Further, the characterization using TLC, FTIR, and LC-MS were performed. The bacteria and genes that produced biosurfactants have been identified via 16S rRNA sequence analysis.

**Results:**

The isolate MW 15 was selected for structural identification given its maximum oil displacement activity, effective surface tension reduction potential, and favourable emulsification index (E_24_) of 51.3 %. The purified biosurfactant from MW 15 exhibited structural similarities to rhamnolipid biosurfactant. The biosurfactant-produced strain was identified as *Stutzerimonas stutzeri* by 16Sr RNA sequencing and the nucleotide sequences were deposited in GenBank with accession number PP779775. Mega 11 software was employed for constructing the phylogenetic tree, and it was confirmed that a gene which produces rhamnolipid (rhlA) was present.

**Conclusion:**

Totally eight bacteria were isolated from petroleum hydrocarbon-contaminated marine harbour water. The isolate *Stutzerimonas stutzeri* showed better production of biosurfactants and biodegradation ability when compared with other biosurfactant-produced isolates. The current investigation promises the use of marine bacteria to produce biosurfactants with biodegradability, less toxicity, and eco-friendly nature.

## Graphical abstract

Fig. A. Graphical Representation of Bioremediation of crude oil and heavy metal pollutants in marine environment by biosurfactant, Rhamnolipid isolated from S*tutzerimonas stutzeri −* MW15.

**Figure:**
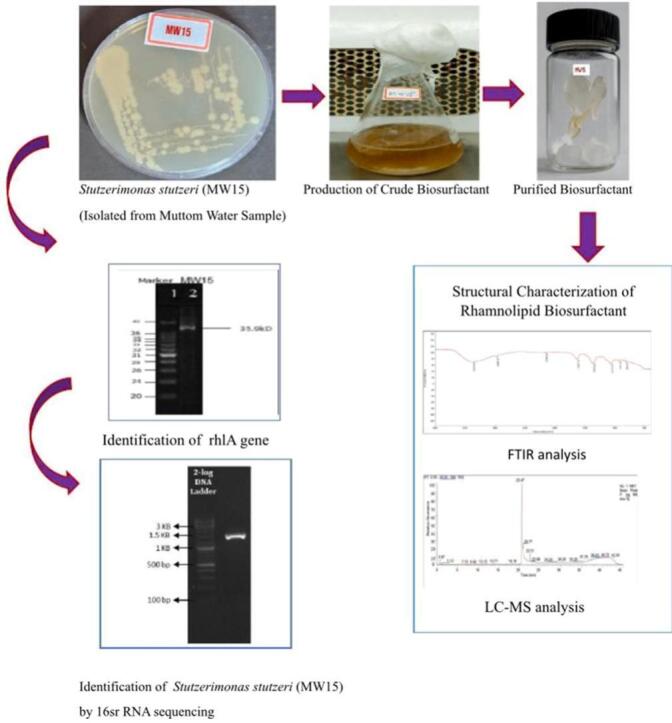

## 1 Introduction

Around 35 million barrels of petroleum are transported annually on average across the world's seas, making the marine ecology susceptible to pollution. The environment and human health are seriously threatened by marine water that is contaminated by petroleum oil and its constituents, particularly hydrocarbons, which are prevalent in industrialized and oil-producing nations worldwide.^1^ Additionally, petroleum contamination is extremely harmful to higher organisms.^2^

Currently, chemically synthesised surfactants are utilised for the degradation of oil contaminants. Chemically produced surfactants aren't recommended as remediating agents given their harmful and persistent properties. Biosurfactants are an alternative for bioremediation of oil spills and crude oil contamination in marine environments. Biosurfactants have significant advantages over chemical surfactants, including biodegradability, lower toxicity, and an eco-friendly nature. Additionally, biosurfactants can be produced from renewable resources and remain effective under various conditions, such as changes in temperature, pH, and salinity.^3^ Rhamnolipids, slichenysins, and surfactins are among the biosurfactants was utilized effectively in environmental remediation.^4^ From a crude oil sample taken in an area damaged by an oil spill, Kim et al.^5^ identified a novel strain of bacteria. Crude oil was removed from surface of contaminated sea sand and degraded by native marine bacteria when biosurfactant-containing remedial agents was incorporated into seawater.^6^ According to Cheong et al.,^7^ biosurfactants may additionally stimulate attachment of bacteria to oil layers, which increases uptake and metabolism of oil hydrocarbons, disrupt oil slicks that are floating on the water's surface, promote oil dispersion, and generate stable emulsion, each of that greatly enhance oil's biodegradation by native marine microorganisms. Based on these factors, biosurfactants having strong potential to be employed to clean up oil spills on shorelines and at sea.^8^

The most extensively researched biosurfactants are rhamnolipids, that are mostly generated by *Pseudomonas aeruginosa* and have a glycolipid-type structure.^9^ This is due to their comparatively high surface activity and high production yield following comparatively brief incubation periods by easily cultivable, well-understood microorganisms. It is a promising sugar-based biosurfactant, used for oil recovery which can be exploited in bioremediation research. In the so-called *Pseudomonas stutzeri* phylogenetic group, also referred to as *P. stutzeri* complex, species that were formerly assigned to the genus *Pseudomonas* have been placed in genus *Stutzerimonas*, that was recommended as potential candidates within *Pseudomonadaceae* family. Research suggests that the Gram negative bacteria *S. stutzeri* have the ability to degrade petroleum hydrocarbons by producing rhamnolipids.^10^

However, the widespread usage of conventional surfactants, which are often sourced from petrochemical sources, needs to be addressed in light of contemporary issues with pollution, health, climate change, and the depletion of fossil fuel supplies. Because of their special qualities and possible uses, biosurfactants and bio-based surfactants have become attractive options (Guzman et al., 2025)^11^. Biosurfactants assist in the emulsification and degradation of oily wastes and can be used in the control of oil pollution. Considering the comparable structural and physical characteristics, are produced from renewable substrates, and have the benefit of degradable efficiency, biosurfactants have the potential to substitute for synthetic surfactants if they become economically competitive. Based on these backdrops, current research is focused on evaluating bioremediation potential of biosurfactants isolated from marine bacteria.

## 2 Materials and methods

### 2.1 Sample collection and isolation of pure culture

The oil-contaminated marine harbour water sample was collected from Chinnamuttom and Muttom harbour in Tamil Nadu, India, in a sterile bottle. To maintain bacterial consortia of water sample, the sample was promptly stored at 4˚C until it was required. Zobell marine agar plates were employed in spread plate approach to isolate pure cultures.^12^ Isolates were selected according to colony morphology on Zobell marine agar plates following 48-hour incubation period. Following screening techniques were employed for determining whether isolates selected produced biosurfactants.^13^

### 2.2 Screening of biosurfactant-producing isolates

In 500 ml Erlenmeyer flask, isolated bacterial strains were cultured aerobically with 100 ml of mineral salt media that contained: 5 g Glucose (C_6_H_12_O_6_), 0.1 g Magnesium sulfate heptahydrate (MgSO_4_·7H_2_O), 0.03 g Ferrous sulfate heptahydrate (FeSO_4_·7H_2_O), 4 g Sodium chloride (NaCl), 0.68 g Potassium dihydrogen phosphate (KH_2_PO_4_), 1.73 g dipotassium hydrogen phosphate (K_2_HPO_4_), 0.02 g Calcium chloride (CaCl_2_·2H_2_O), 1 g Ammonium nitrate (NH_4_NO_3_), and 1.0 % crude oil (W/V).

Flasks with sterilized mineral salt media were infected with isolated bacteria and maintained in a shaker at 200 rpm and 30˚C for 7 days. After 7 days of incubation, culture broth was centrifuged at 6000 rpm at 4˚C for 15 min and filtered through Millipore 0.45 µm filter paper. Direct hemolysis screening was performed on these isolated isolates.^14^ Oil displacement assay (Masaaki  et al., 2000^15^), drop collapse assay (Bodour and Miller, 1998^16^), and tilted glass slide assays assessed strains' biosurfactant production.

### 2.3 Biodegradation screening by emulsification activity

After 48 h of incubation, pure culture colonies were suspended in test tubes with 2 ml mineral salt media and 2 ml petroleum. After 1 min of high-speed vortexing, mixture left for 24 h. Formula for calculating E24^17^:

$$Emulsification\;Index\;\left(\;E24\right)=\frac{Height\;of\;emulsified\;layer}{Total\;height\;}\times 100$$

### 2.4 Effect of biosurfactant on heavy metal removal

Biosurfactant was employed to remove lead, mercury, cadmium, and arsenic. Prepared and sterilized nutrient broth medium with lead, mercury, cadmium, and arsenic salts. Lead, mercury, cadmium, and arsenic salts was added at 20, 40, 60, 80, and 100 mg/l, respectively. The pH of medium was adjusted to 7 and sterilized in an autoclave at 15 Lbs pressure for 15 min. Then extracted biosurfactants (about 50 µL) was inoculated into the medium and incubated for 24, 48, 72 and 96 hrs at 30˚C to study the degradation effects. Sterilized nutrient broth was maintained as control. After incubation the tubes were analyzed for the concentration of metals present after treatment in UV–Vis spectrometer.^18^

### 2.5 Biosurfactant extraction from the selected bacterial strain

Biosurfactant extraction was conducted on the MW15 strain post-screening. A shaking incubator at 120 rpm incubated 100 ml of optimum medium and mineral salt medium at 25 °C for 7 days with MW15 inoculation. Centrifugation at 5000 rpm at 4 °C for 20 min removed cells. The supernatant was taken, and the pH of the supernatant was adjusted to 2, using 1 M H_2_SO_4_. After adding equal volumes of chloroform: methanol (2:1), mixture was shaken well and left overnight to evaporate. After evaporation, the white-coloured sedimented biosurfactant was taken for further analysis.^19^

### 2.6 Dry weight determination of biosurfactant

Weight was measured on sterile petri plate. Plates were then poured with white sediment. Dried for 30 min at 100 °C in hot air oven. Upon drying, plates were weighed, and then following formula was employed to determine dry weight of biosurfactants^19^:

Dryweightofbiosurfactant=Weightoftheplatesafterdrying-Weightoftheemptyplate

### 2.7 Separation of biosurfactant by Thin-layer chromatography (TLC)

TLC was performed according to Rashedi et al.^20^ procedure. 10 µl of biosurfactant has was applied at point origin close to bottom of TLC (silica gel 60G) plate after 10 mg of biosurfactant was dissolved in 15:35 v/v chloroform:methanol. The plate then allowed to air dry in a fume cupboard after being produced in a solvent solution. The plate was sprayed uniformly with water or sulphuric acid solution, and it was then placed in an oven at 110˚C for 20 min to observe spots. At preparative scale, spots were removed by scraping the silica from the plate into a flask. Extract diluted in chloroform: methanol (2:1) for analysis.

### 2.8 Characterization of biosurfactant

#### 2.8.1 Biochemical characterization

Following Sawhney et al.'s^21^ standard approach, biochemical assays was employed for identifying biomolecules in crude biosurfactant. Anthrone test determined carbohydrate moiety in biosurfactant sample, ninhydrin test detected amino acids and associated polymer proteins, and saponification test measured lipid content.

#### 2.8.2 Spectral analysis (FTIR)

FTIR spectroscopy was employed to examine biosurfactants' functional groups. IR Prestige- 21 Fourier Transform Infrared spectrophotometer was used for determining functional group of the biosurfactant by the KBr pellet method.

### 2.9 LC-MS analysis

Purified product biosurfactant mixtures have was separated and LC-MS (Agilent Technologies 1260 Infinity LC and 6410 Triple Quad MS, USA) was employed for determining different congeners (structural analogs). Biosurfactant sample was diluted in methanol and inserted into ZORBAX C18 column (2.1 × 50 mm^2^) with 2 µl aliquot. A 0.20 mL/min flow rate was maintained for LC. Column mobile phase was acetonitrile/water gradient (10–90 %) with 0.01 % formic acid. Agilent software analyzed positive ion ESI-MS data. With 135.0 V fragment of voltage, full scan data was collected from *m*/*z* 150–2000.

## 3 10 biosurfactant-producing isolate “genomic DNA isolation and identification

Genomic DNA was isolated and purified from“ selected biosurfactant-producing bacteria MW15 based on the method of Sivasankari et al.^22^

### 3.0.1 Detection of biosurfactant-producing genes using Real-Time PCR

Biosurfactant-producing genes detection in *S. stutzeri* using real-time PCR (qPCR) is highly sensitive and specific technique. Biosurfactant-producing rhamnosyl transferase (*rhl A*) gene responsible for rhamnolipid biosynthesis, can be targeted for detection and quantification. Rhamnosyl transferase (*rhlA*) gene was amplified by rhlA F (ATGCGGCGCGAAAGTCTGTTG) and rhlAR (TCAGGCGTAGCCGATGGCC) primers (Kumar et al., 2008)^23^. PCR was performed with 1 μL of DNA template from selected biosurfactant manufacturer. To prepare primers (0.2 μM), include 10 × reaction buffer, 1.5 mM MgCl_2_, 200 μM dNTPs, and 1U Taq DNA polymerase in Thermal Cycler (BioRad). DNA was amplified at 95 °C for 2 mins, followed by 40 cycles of denaturation (15 s), annealing (30 s), extension (30 s), and final extension (10 min) at 72 °C (Pacwa-Płociniczak et al., 2014)^24^.

### 3.0.2 16s rRNA sequencing and phylogenetic analysis

After purification of genomic DNA from MW15 strain, the 16S rRNA amplification was done using universal primers 16S-RS-F (CAGGCCTAACACATGCAAGTC) and 16S-RS-R (GGGCGGWGTGTACAAGGC). PCR amplification was carried out using 2X Phire Master Mix (5 µl), forward primer (0.25 µl), Reverse primer (0.25 µl), and 1 µl of sample DNA, under the following parameters: 95 °C for 5 min followed by 35 cycles of 95 °C for 30 sec, 60 °C for 40 sec, and 72 °C for 60 sec as well as a final extension at 72 °C for 7 min. The PCR products were checked in 1.2 % agarose gels.

NCBI genbank database was employed to perform BLAST using 16S rDNA gene sequence. Clustal W, a multiple alignment software program, was employed to align first ten sequences according to maximum identity score. Phylogenetic tree was constructed by neighbor joining and Kimura 2-parameter method and a distance matrix was generated. Employing MEGA 11 software, bootstrap analysis of 1000 sets was conducted for evaluating topologies. Through BANKIT sequence submission tool, isolate's assembled complete 16S rRNA sequence was submitted to NCBI Gene Bank (Narasimman *et al.,* 2021)^25^.

## 4 Results and discussion

### 4.1 Enrichment and isolation of the bacterial isolates

Samples of marine harbour water contaminated with petroleum hydrocarbons were collected from Muttom and Chinnamuttom in Tamil Nadu, India. The sample was kept for 7 days at 200 rpm in Mineral Salt Medium (MSM) containing 1 % crude oil as only source of carbon. After being serially diluted, enriched samples were spread-plated onto ZMA that was amended with crude oil. For further studies, 30 different colonies were selected according to their morphology and then subjected to additional biosurfactant screening.

### 4.2 Screening of biosurfactant production

Among 30 isolates, 4 bacterial strains isolated from Chinna Muttom water sample which are named as CW4, CW10, CW11, CW13 and 4 bacterial strains isolated from Muttom water sample which are named as MW6, MW7, MW9, and MW15 were showed the best activity in all screening methods. The results demonstrated that strain MW15 had highest activity for emulsification activity (51.3 %) and oil displacement tests. Hemolytic activity was positive among 8 bacterial isolates. For production of biosurfactants, blood hemolysis is a crucial initial screening technique. While some microbial products, including virulence factors, lyse blood agar, less diffusible biosurfactants don't lyse blood cells.^26^

Surface and wetting activities are indicated by drop collapse and oil spreading tests (Youssif *et al*., 2004)^27^. In oil spreading test all the eight isolates displaced the oils, and the results were shown in Table 1. Among all isolates MW15 showed high activity against all the tested oils. In drop collapse test CW13, MW6, MW7, MW9 and MW15 showed positive results and other isolate such as CW4, CW10 and CW11 did not show any activity. Surfactants, assisting in the cells' adhesion to oil droplets, are typically produced when bacteria grow on hydrocarbons.^28^ In tilted glass slide test except CW4, CW10 and MW6 all other isolated strains showed positive activity.

**Table 1:** Screening of biosurfactants for selected strains using different methods.

Sample	Hemolytic activity	Oil spreading (diameter)					Drop collapse	Tilted glass slide
		Diesel	Vegetable oil	Kerosene	Petrol	Crude oil		
CW4	++	++	++	+	++	+	−	−
CW10	+	+	+	+	+	+	−	−
CW11	+	++	−	+	++	−	−	++
CW13	++	+	++	++	++	+	+	+
MW6	+++	+	+	−	++	+	+	−
MW7	+++	++	++	++	++	++	+++	+++
MW9	++	+	+	++	+	++	++	+
MW15	+++	+++	++	+++	+++	+++	+++	+++

+++ − >5cm, ++ − a-3 cm, + −<1cm, in oil displacement: +++ − highly positive, ++ − moderate positive, + − weak positive, − − no displacement.

The emulsifying activity of all strains was subjected to different types of hydrocarbons such as diesel oil, crude oil, n-hexadecane, n-hexane, kerosene, and xylene (Table 2). All the isolates showed the minimum activity, and the MW15 isolate showed the highest activity in all hydrocarbons especially 51.3 % against crude oil. There is no activity was reported in control. Bioemulsifier productivity is determined by the emulsification index.^29^ Selecting an effective biosurfactant producer may be accomplished by examining emulsification units .^14^ This serves as one of the crucial and effective assays for screening biosurfactant producers.

**Table 2:** Emulsifying activity of selected strains in different types of hydrocarbons such as Diesel oil, Crude oil, n-hexadecane, n-hexane, kerosene, and xylene.

Samples	Hydrocarbon (E_24_/%)					
	Diesel oil	Crude oil	n-Hexadecane	n-Hexane	kerosene	xylene
CW4	41.5	40.2	41.3	43.2	43.5	40.5
CW10	34.2	33.5	31.5	29.2	31.6	36.8
CW11	32.7	30.4	33.1	33.5	36.6	30.8
CW13	44.3	42.1	41.5	44.4	40.2	41.3
MW6	36.4	34.2	32.2	30.4	35.5	34.1
MW7	51.2	50.3	48.7	50.8	49.6	52.1
MW9	43.4	41.3	40.1	47.5	42.6	46.3
MW15	52.6	51.3	50.5	48.2	51.8	48.8

^30^ demonstrated that *Stutzerimonas stutzeri* has high biodegradation efficiency, degrading up to 84 % of crude oil in bioremediation studies, attributed to its robust rhamnolipid production. Its surfactants have low critical micelle concentrations (CMC), indicating high surface activity. Bacillus subtilis showed 70–80 % degradation of hydrocarbons.^31^ However, its lipopeptides are less versatile than rhamnolipids for certain industrial applications. *S. stutzeri* performs comparably to *P. aeruginosa* and outperforms *B. subtilis* in biodegradation efficiency, making it a strong candidate for environmental applications like oil spill remediation.

### 4.3 Heavy metal degradation by biosurfactant

The heavy metal treated with biosurfactant showed a reduction in heavy metal concentration. The percentage of remediation varied with the concentration of biosurfactant. When the concentration of the biosurfactant was increased to 100 mg/l, higher remediation efficiencies were obtained and it was noted that the reduction of 60.4 % Pb, 59.8 % As, 55.7 % Cd and 52.5 % Hg (Table 3). Current research reported the bioremediation of heavy metals by the action of biosurfactant in a concentration-dependent manner. The results were in agreement with studies in bioremediation of heavy metals. Based on research, it was postulated that the removal of these heavy metals by the biosurfactants by means of sorption, complexation, precipitation and surface adsorption.[32], [33]

**Table 3:** Biodegradation of heavy metal degradation by biosurfactant isolated from MW15.

Concentration of heavy metal (mg/L)		Incubation time (hours)			
		24	48	72	96
		Heavy metal degradation (%)			
Lead	20	26.7	34.8	41.8	49.3
	40	29.8	36.8	43.7	51.2
	60	32.5	39.2	49.6	55.8
	80	37.9	42.5	50.8	54.7
	100	45.4	50.1	57.6	60.4
Mercury	20	20.2	25.4	23.6	32.5
	40	24.5	29.7	32.5	37.5
	60	27.6	31.2	35.6	44.7
	80	32.5	35.7	39.8	48.9
	100	39.7	42.3	46.2	52.5
Arsenic	20	19.2	24.9	33.7	40.2
	40	25.4	29.8	37.5	43.1
	60	32.8	37.8	44.2	50.2
	80	37.9	41.3	49.2	55.7
	100	42.1	46.1	52.6	59.8
Cadmium	20	21.5	25.9	29.7	35.7
	40	25.6	31.2	37.3	41.6
	60	30.2	35.7	40.5	46.8
	80	34.8	39.8	44.2	49.7
	100	40.2	45.8	49.2	55.7

### 4.4 Biosurfactant extraction and dry weight determination

From the biosurfactant-producing bacterial strain MW15, the biosurfactant was extracted in a mineral salt medium, and the white sediment of the biosurfactant was weighed as 6.69 g per 100 mL of medium.

### 4.5 Isolation and purification of biosurfactant by thin layer chromatography

TLC purified biosurfactant that was extracted. When 5 % sulphuric acid reagent sprayed on TLC plate containing crude biosurfactant, it appeared yellow. Based on the R_f_ value and colour of the spot, the biosurfactant was identified as rhamnolipid (R_f_ = 0.92).

### 4.6 Characterization of biosurfactant

#### 4.6.1 Biochemical analysis

Lack of ruhemann's purple complex development in Ninhydrin test during biochemical investigation of biosurfactant indicates that there are no amino acids or proteins in biosurfactant. Carbohydrates present in biosurfactant was demonstrated by blue-green color development that was observed in anthrone test. The presence of lipids is demonstrated in saponification test when lipids in biosurfactants are saponified by NaOH. The presence of biosurfactants is confirmed by result, indicating that crude biosurfactant produced from bacterial MW15 consists of lipid and sugar molecules.

#### 4.6.2 Spectral analysis (FTIR)

**Fig. 1:**
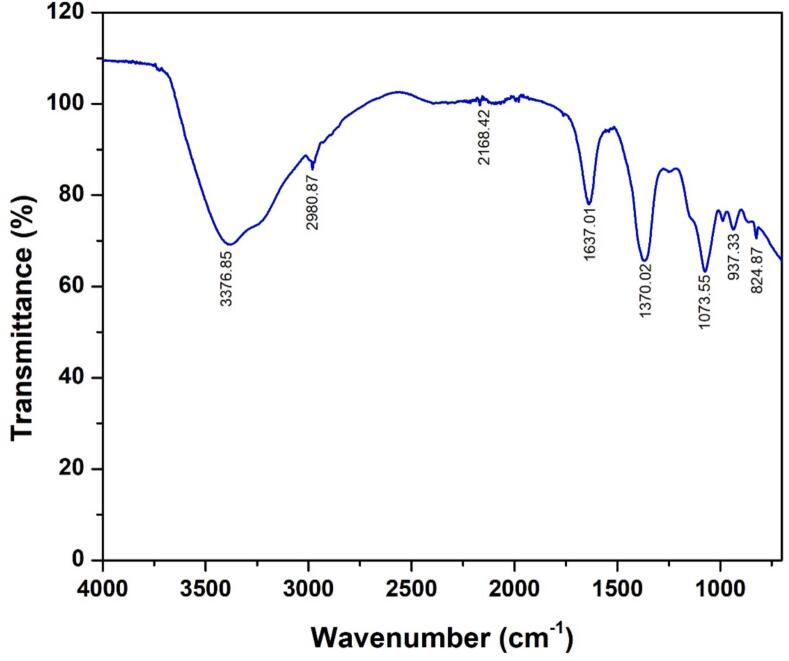
FTIR spectral analysis of rhamnolipid biosurfactant from MW15.

A prominent absorption band was observed at 3376.85 cm^−1^ and 2980.87 cm^−1^ in crude biosurfactant spectrum of strain MW15 (Fig. 1; Table 4). This was generated by O–H bonding and C–H stretching, respectively. At 824.87 cm^−1^, C–H stretching was additionally detected. N-O stretching and C-O stretching were found at 937.33 cm^−1^ and 1073.85 cm^−1,^ respectively. CH_2_-CH_3_ groups and C–C stretching were found at 1370.02 cm^−1^ and 1637.01 cm^−1,^ respectively. The absorption at 2168.42 cm^−1^ was because of the stretching of the Si-H silane group. Absorption bands pattern observed in FTIR analysis indicates that the polysaccharide substance is present in the biosurfactant. From this, it was confirmed that the isolated biosurfactant was glycolipid in nature. The similar characteristics of rhamnolipid were confirmed by Abbasi et al.^34^ and Rath et al.^35^ Beema Kumari *et al.* (2023)^36^ isolated and characterized biosurfactant from the Stutzerimonas stutzeri (LOBP-19A) present in hydrocarbon-contaminated soil and reported the peak between 3253.10 cm^−1^ to 719.2 cm^−1^. Putri and Hertadi (2015)^37^ isolated and characterized the biosurfactant using FTIR analysis *P. stutzeri* BK-AB12 and reported the peaks between 3427 and 1745 cm^−1^ representing vibration of O–H, CH3, CH2 aliphatic groups, C=O of ester group and –C–O– of ether functional group, showing the characteristics nature of rhamnolipid which shows the high similarity as in case of” current research.

**Table 4:** Wavenumber and functional groups of FTIR spectroscopy investigation of MW 15.

S. No	Peak	Characteristic Absorptions (cm^−1^)	Possible functional Group	Class
1	824.87	500–1000	C–H out of plane	Aromatic
2	937.33	500–1000	=NOH (N-O)	Misc
3	1073.85	1000–1500	C-O stretch	Ether
4	1370.02	1000–1500	CH2 and CH3	Alkanes
5	1637.01	1500–2000	C–C stretch	Alkenes
6	2168.42	2000–2500	Si-H silane	Misc
7	2980.87	2500–3000	CH stretch	Alkanes
8	3376.85	3000–3500	O–H bonded	Phenols

#### 4.6.3 LC-MS analysis

**Fig. 2:**
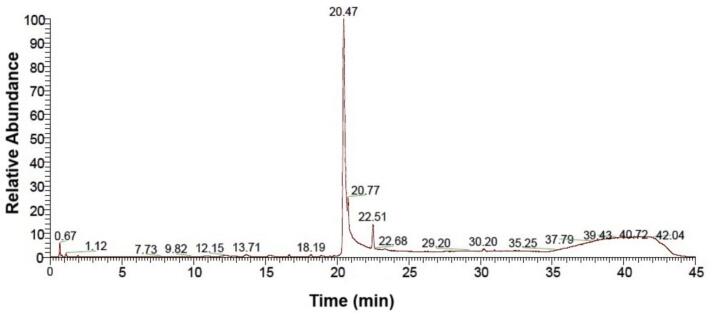
LC-MS analysis of rhamnolipid biosurfactant from MW15.

Purified biosurfactant was subjected to LC-MS analysis in positive ion mode to identify its structural elements (Abdel-Mawgoud *et al*., 2010)^38^. The LC MS analysis of samples isolated from *Stutzerimonas stutzeri* revealed a relative abundance of 20.47 % rhamnolipid. The results suggest that the rhamnolipid constitute the significant portion of the biosurfactant produced by this bacterium (Fig. 2).

#### 4.6.4 Morphological and biochemical characterization of isolated bacterial strain

The colony of isolate MW15 showed rod and pale colour, medium in size and the cells were gram-negative with rod-shaped and motile in their cell characters. In the biochemical characterisation, the isolate showed the positive results for catalase, oxidase, citrate utilization, and nitrate reduction tests and negative for glucose fermentation tests.

### 4.7 Detection of biosurfactant-producing genes using Real-Time PCR

**Fig. 3:**
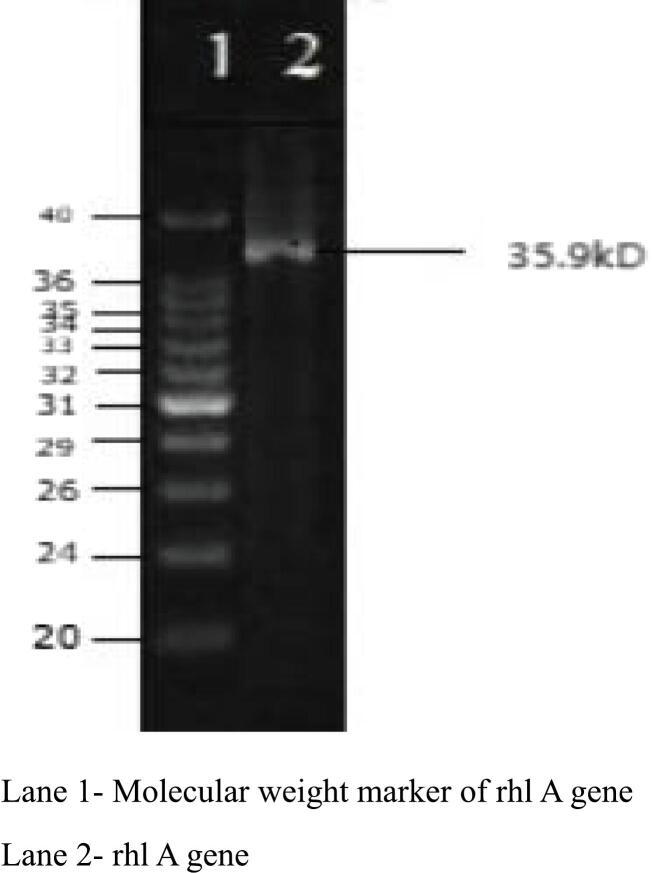
Detection of rhlA gene by Real – Time PCR.

The screening of biosurfactant-producing genes, specifically rhlA, is crucial in identifying bacterial strains capable of producing biosurfactants, which have significant industrial and environmental applications. In this study, we found that the isolates tested were positive for the rhlA, genes (Fig. 3). These findings are discussed in the context of the rhamnolipid biosynthesis pathway and their potential applications. The rhlA gene encodes 3-(3-hydroxyalkanoyloxy)-alkanoic acid synthase, which is involved in synthesis of lipid moiety of rhamnolipids. The detection of rhlA suggests that isolates have potential to produce the lipid backbone necessary for the synthesis of rhamnolipids. Previous studies have shown that strains positive for rhlA typically exhibit efficient biosurfactant production, and are valuable for applications in bioremediation, enhanced oil recovery, and microbial control of hydrophobic pollutants,^39^ Deosthali *et al.,* 2024)^40^. The detection of rhlA genes in the isolates suggests that these bacteria are capable of producing rhamnolipids, which are highly efficient biosurfactants with numerous applications. These findings are significant in the context with the findings of,^41^ where the study reported disperants and rhamnolipid enhanced oil biodegradation in coastal sediments.

### 4.8 Identification and phylogenetic analysis of biosurfactant-producing bacterial strain

**Fig. 4:**
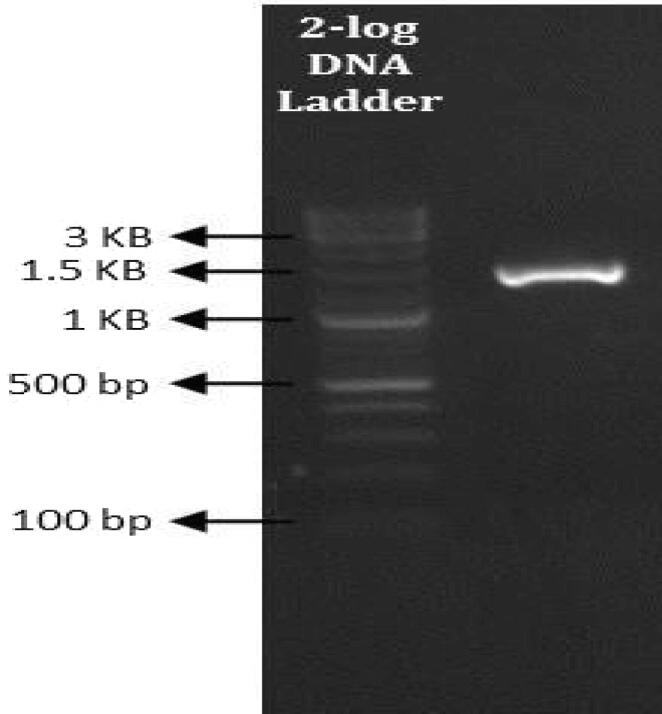
16sr RNA PCR amplification of Stutzerimonas stutzeri bacterial DNA.

**Fig. 5:**
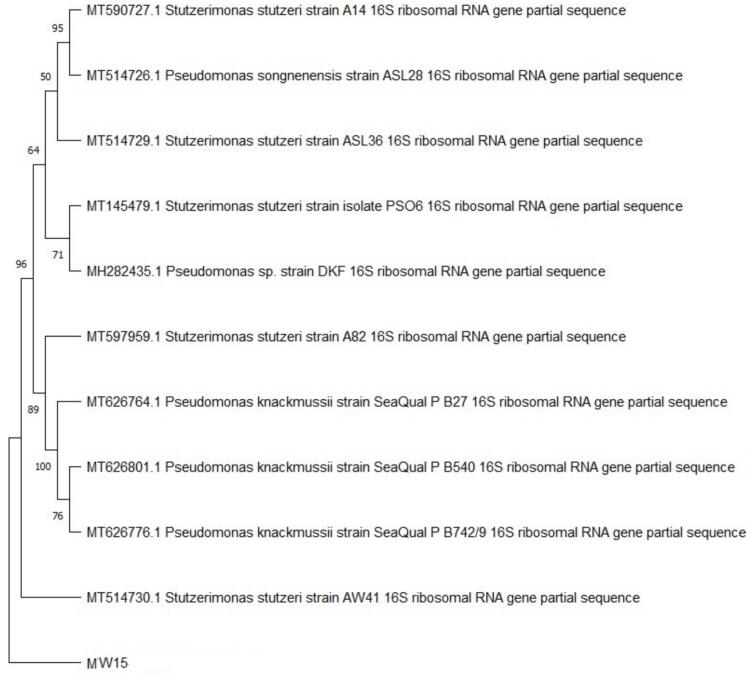
Phylogenetic tree of isolate MW15 based on 16S rRNA sequences.

The phylogenetic analysis of MW15 strain revealed *S. stutzeri* by 16s rRNA sequencing. The 16s rRNA amplification of *S. stutzeri* produced about ~1.5 k bp amplification (Fig. 4), and the phylograms were generated with 16s rRNA gene sequence of very similar sequences of other bacteria of the NCBI database (Fig. 5). Gene Bank received assembled 16S rRNA sequence of *S. stutzeri,* that was assigned accession number PP779775. Oil sludge from Abadan refinery in Iran was utilized for isolation and identification of *P. stutzeri* (AOR1) strain. Afifi et al.^42^ claimed that this species may recover oil by producing “biosurfactants. *P. stutzeri* strain (HS-D36) (PS2) was additionally isolated from petroleum sludge and crude oil-contaminated soil by Mohd Kamil *et al*. (2012)^43^ and Guellal et al.^44^″. It was determined that this strain can produce biosurfactant for improving biodegradation of phenanthrene. Rath et al.^35^ reported that *Pseudomonas* exhibited strong emulsifying, foam-forming, and surface tension properties in petroleum-contaminated environments. *P. stutzeri* is extensively isolated from the environment and utilised in oil biodegradation.^42^ Pseudomonadaceae family's proposed genus *Stutzerimonas* includes species from *P. stutzeri* phylogenetic group, often referred to as *P. stutzeri* complex.^45^ The amphiphilic structure allows them to reduce surface tension at interfaces, facilitating the emulsification of hydrophobic compounds. They play a crucial role in various applications, including bioremediation, agriculture, and biomedical fields, leveraging their capacity to reduce surface tension and emulsify hydrophobic substances However, limitations like production costs and scalability hinder their widespread adoption.

*Stutzerimonas stutzeri* is a highly versatile, utilizing low-cost, renewable substrates like agro-industrial wastes (molasses, cassava wastewater, dairy waste, vegetable oil waste).^30^ This reduces substrate costs, a major economic hurdle in biosurfactant production. *Stutzerimonas stutzeri* is a practical candidate for biosurfactant production, offering competitive yields, excellent substrate flexibility, and high biodegradation efficiency. Its non-pathogenic nature and ability to use low-cost substrates like agro-industrial wastes make it economically promising, though it trails *P. aeruginosa* in yield and scale-up readiness.

## 5 Conclusion

Marine microorganisms were utilized to generate biosurfactants and bioremediate oil-contaminated sites in limited experiments. Current research confirms that *S. stutzeri* (MW15) is a potential source for rhamnolipid biosurfactant production and “bioremediation applications, particularly in the remediation of crude oil contamination sites”. Further optimization studies can enhance the biosurfactant-producing capacity of this strain. Research results contribute to the growing body of research on the application of biosurfactants for environmental remediation. Hence, this microbial biosurfactants will be an environmentally friendly alternative to synthetic surfactants because of their biodegradability and low toxicity, which enhance nutrient availability, inhibit the growth of pathogens, modulating microbial communities and enhance the nutrient availability in soil.

## Ethical statement

Not Applicable. No human participants or subjects or animals were involved in this study.

## Author contribution

Sony J B - Original article writing, Manjusha W A- Supervision, reviewing and editing. Sangeetha VS- Editing, Salom Gnana Thanga Vincent- Editing, Citarasu T.- Reviewing and editing, Anusha JR- Formal analysis and editing.

## Funding

No Funding received to carry out this research.

## Declaration of competing interest

The authors declare that they have no known competing financial interests or personal relationships that could have appeared to influence the work reported in this paper.
